# Intestinal neuronal dysplasia presenting as psoas abscess: A case report

**DOI:** 10.3389/fsurg.2022.957730

**Published:** 2022-11-01

**Authors:** Bing Bing Ren, Bo Zhang, Shu Xian Chen, Hong Qiu Han, Da Qing Sun

**Affiliations:** ^1^ Department of Pediatric Surgery, General Hospital, Tianjin Medical University, Tianjin, China; ^2^ Department of General Surgery, General Hospital, Tianjin Medical University, Tianjin, China

**Keywords:** intestinal neuronal dysplasia, psoas abscess, inflammatory bowel disease, case report, constiption

## Abstract

**Background:**

Intestinal neuronal dysplasia (IND) is a rare condition mainly affecting the children. Constipation and abdominal distension have been reported as common manifestations. In addition, the reports about adult cases are scarce.

**Case report:**

A 31-year-old man presented with pain in his left hip and intermittent fever for 1 month. The whole abdomen CT and pelvic contrast-enhanced MRI revealed a left psoas abscess (PA). The patient has been given anti-infective treatment and underwent CT-guided drainage of left PA with a temporary drain. But the patient's condition did not improve significantly. Then, the colonoscopy revealed that it may be the PA secondary to inflammatory bowel disease. But the pathology was not in line with inflammatory bowel disease. We finally performed an ileostomy surgery and took the whole layer of intestinal wall for biopsy. The pathological result revealed that a large number of proliferative ganglion cells and circuitous hyperplastic nerve fibers were found in the submucosa and muscular layer of the intestinal wall. Given pathological results and clinical manifestations, the patient was diagnosed with IND-B.

**Conclusion:**

In this case, we first report an extremely rare case of adult IND manifesting as PA. So, this unusual case provides a new supplement to adult cases of IND.

## Introduction

Intestinal neuronal dysplasia (IND) is a rare anomaly of the enteric nervous system, with an estimated incidence of approximately one in 7,500 newborns (1). This disorder is a frequent cause of gut dysmotility and pseudo-obstruction which shows the clinical features similar to Hirschsprung's disease (HD). But, IND is a distinct clinical entity genetically different from HD (2). Due to IND mainly affecting children, few adult cases have been reported. Here, we report an extremely rare case of adult IND with psoas abscess (PA) as the initial symptom.

## Case presentation

A 31-year-old man came to our hospital with left hip pain and intermittent fever for 1 month. For nearly a year, the patient occasionally has abdominal distention which alleviated after defecation. The frequency of defecation was about once every two days. The mass was seen as a reddish skin color around the anterior superior iliac spine which was painful and palpated.

## Diagnostic assessment

**Figure 1:**
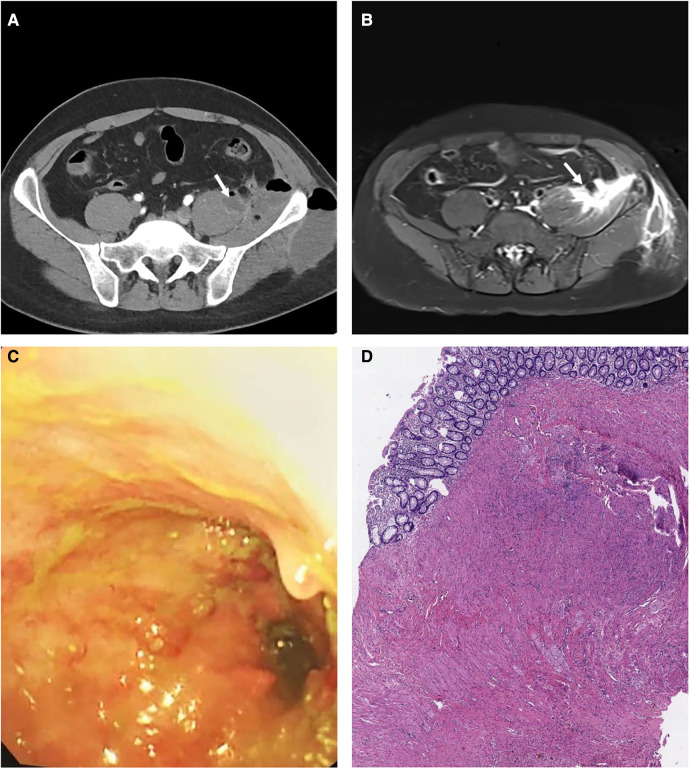
The abdominal CT showed patchy low-density shadow of the left pelvic wall soft tissue (**A**). The pelvic MRI showed that the lower part of the left psoas major muscle, the left pelvic wall and the left buttock were thickened with multiple high signal shadows (**B**). Colonoscopy showed multiple filling defects and local lumen stenosis in the terminal ileum, ileocecum and the beginning of the ascending colon (**C**). The pathological results revealed that a large number of proliferative ganglion cells and circuitous hyperplastic nerve fibers were found in the submucosa and muscular layer of the colon wall (**D**).

The laboratory examination revealed white blood cell 13.25 × 10^9^/L (3.5–9.5), neutrophil percentage 85% (40–75), platelet 420 × 10^9^/L (125–350), C-reactive protein 82.6 mg/L (0–10) and erythrocyte sedimentation rate 48 mm/h (0–20). The tumor-related biomarkers (AFP, CEA, PSA, CA19–9, ferritin), serum tuberculosis antibodies and T-cell spot test for tuberculosis infection (T-TB.Spot) were within normal limits. The whole abdomen CT and pelvic contrast-enhanced MRI were performed. The imaging revealed a left PA (Figures 1A,B) Colonoscopy revealed mucosal stiffness and multiple polyps on the ascending colon (Figure 1C). Then, the mucosal biopsy of ascending colon was performed. To sum up, we think that it may be the PA secondary to inflammatory bowel disease. But pathology was not in line with the typical manifestations of inflammatory bowel disease.

## Treatment

Since admission, the patient was given piperacillin sodium plus tazobactam for anti-infective treatment, but the patient's condition did not improve significantly. Then, the patient underwent CT-guided drainage of left PA with temporary drain placement and drained 450 ml pus. The patient still had intermittent pain and fever. We finally decided to perform a laparoscopic exploration. During the operation, we found the sigmoid colon with slight expansion and severely adhering to the surrounding tissue. Later, we performed a temporary ileostomy surgery and tried to take the whole layer of intestinal wall (descending colon, sigmoid colon about 0.5 cm in diameter) for biopsy. In addition, the primary closure was done after full thickness biopsy. The patient recovered well and was discharged two weeks after the operation. Moreover, followed up for 6 months after operation, the fever and pain in his left hip was no recurrence without specific treatment. The temporary stoma was scheduled to be closed one year after surgery.

## Histopathology

The result of mucosal biopsy was scattered infiltration of lymphocytes, plasma cells and eosinophils in the colonic mucosa. The Haematoxylin and Eosin staining technique has been used for hystopathological diagnosis. The pathological results revealed that a large number of proliferative ganglion cells and circuitous hyperplastic nerve fibers were found in the submucosa and muscular layer of the intestinal wall (Figure 1D). Given pathological results and clinical manifestations, the patient was diagnosed with IND-B.

## Discussion

Swiss pathologist Meier-Rule first proposed the pathological phenomenon of colonic neuronal dysplasia in 1971 (3). It is classified into two clinical and histologically subtypes as types A or B. IND type B (IND-B), which comprises >95% of IND cases, is a pathological entity of the group of gastrointestinal neuromuscular diseases characterized by hyperplasia of the submucosal nerve plexuses (4) and presents as chronic constipation usually during childhood (5). In addition, the etiopathogenesis of IND-B is widely debated. It is mainly recognized as genetic alterations resulting in intestinal neuronal system development disorder (6, 7). However, IND-B can also be understood as a secondary phenomenon due to congenital intestinal obstructions or local inflammatory processes (8).

There had been several adult cases reported in the past few years. In addition, constipation and abdominal distension were a common manifestation in reported adult cases (9, 10). Referring to the relevant literature, the adult IND with PA as the initial symptom is reported for the first time in our case. The PA is an infectious disease with nonspecific clinical presentation which frequently leads to diagnostic difficulty. The PA is mostly secondary abscess. The most common etiologies of PA were vertebral osteomyelitis, colorectal cancer, gastrointestinal tract infection and Crohn's Disease (11, 12). Therefore, when the patient was admitted to the hospital, we first measured the tumor-related biomarkers, serum anti-tuberculosis antibodies and T-cell spot test for tuberculosis infection (T-TB.Spot). The above indexes all indicated negative results. And combining with imaging examinations, some possible diseases such as tuberculosis, tumor or vertebral osteomyelitis were excluded. In addition, we also took into account the diagnosis of Crohn's disease. In our case, both colonoscopy and barium enema revealed marked stenosis of colon, but the result of mucosal biopsy did not conform to the pathological manifestations of Crohn's disease. The above imaging and related examination results made the diagnosis more difficult, coupled with long-term anti-infective treatment did not have a good effect. Finally, we decided to explore the abdominal cavity and perform full-thickness biopsy of colon wall. Pathologic examination of the specimen showed that there were a large number of proliferated ganglion cells and nerve fibers in the submucosa and muscular layer of the colon wall, which can be considered as an important reference index in the process of diagnosis. Combining the clinical manifestations with the results of pathological and laboratory investigations, the diagnosis of IND-B was established.

Due to the rarity of adult cases and the non-specificity of symptoms, the diagnosis of adult IND is more difficult than that of infants. And early diagnosis remains a great challenge for IND. The auxiliary diagnostic methods include barium enema, anorectal manometry and rectal mucosa biopsy. However, the histological examination remains the gold standard of the diagnosis. Usually, it is necessary to include a sufficient amount of submucosa in the suction biopsy specimens. In this case, it is worth noting that the rectal mucosal biopsy of the patient was negative. Thus, full-thickness biopsy of colon wall can be considered as an important reference index in the diagnosis of IND.

According to the latest report, blood Sox 10 promoter methylation can be used as a noninvasive and efficient diagnosis method for IND (13). Recently, an endoscopic device has been developed to obtain full-thickness biopsies from the bowel wall without laparotomy and anesthesia (14). It is a promising minimally invasive procurement of intestinal full-thickness biopsies for the diagnosis of intestinal neuropathies.

## Conclusion

In conclusion, for patients with PA, physicians should consider IND as a possible diagnosis after excluding other more common causes. So, this unusual case of psoas major abscess provides a new supplement to adult cases of IND. More importantly, a non-invasive diagnostic method with a high degree of accuracy needs to be developed. No matter for the diagnosis and treatment of IND in infants and adults, further exploration is needed and attention should be paid to individual treatment.

## Data Availability

The original contributions presented in the study are included in the article/Supplementary Material, further inquiries can be directed to the corresponding author/s.
